# The International Risk Governance Council: Reflections on a 20‐Year Experiment in Support of Improved Risk Governance

**DOI:** 10.1111/risa.70117

**Published:** 2025-10-05

**Authors:** M. Granger Morgan, Marie‐Valentine Florin, Igor Linkov, Kenneth A. Oye, Arthur C. Petersen, Ortwin Renn, Jonathan B. Wiener, Lan Xue

**Affiliations:** ^1^ Department of Engineering and Public Policy Carnegie Mellon University Pittsburgh Pennsylvania USA; ^2^ IRGC Secretariat (ret.), École Polytechnique Fédérale de Lausanne (EPFL) Lausanne Switzerland; ^3^ US Army Engineer Research and Development Center Concord Massachusetts USA; ^4^ Department of Political Science Massachusetts Institute of Technology Boston Massachusetts USA; ^5^ Department of Science Technology Engineering and Public Policy University College London London UK; ^6^ Institute for Advanced Sustainability Studies (ret.) Potsdam Germany; ^7^ Duke University School of Law Environment, and Public Policy and Duke Center on Risk Durham North Carolina USA; ^8^ Schwarzman College and School of Public Policy and Management Tsinghua University Beijing China

**Keywords:** international collaboration, IRGC, risk governance

## Abstract

The International Risk Governance Council (IRGC) was a nonprofit foundation, based first as an independent, freestanding Swiss foundation in Geneva from 2003 to 2012, and then affiliated with École Polytechnique Fédérale de Lausanne in Lausanne from 2012 to 2023. IRGC's mission was to identify and improve the governance of emerging and systemic risks that have, or could have, impacts on human and environmental health, the economy and society, and overall sustainability. In this paper, we recount IRGC's history, describe its many reports, workshops, and conference activities (including tables referencing the many published products), and provide six brief case histories of accomplishments and insights on work IRGC has done on solar radiation management, small modular reactors, synthetic biology, autonomous vehicles, resilience and systemic risks, and international comparison of risk governance. The paper concludes with some brief observations about the impact of IRGC's work and notes the continuing need for a neutral convening entity that can perform a role similar to that of IRGC.

## 1 Background and Brief History

At the beginning of the century, a small group of leading corporate, government, and academic leaders held a series of informal discussions at the Engelberg Forum in Switzerland and identified the need for an organization that could work to improve the governance of risks.^1^ It was the collective judgment of that group that many risks could not be simply “managed” or “controlled” because handling them requires a broader “governance” approach.

As a follow‐on to these discussions, the International Risk Governance Council (IRGC) was created in Geneva. The Secretariat for Education, Research, and Innovation (SERI)^2^ of the Swiss Federal Government assisted in creating the organization. Although it is in the heart of Europe, Switzerland is not a member of the European Union. It has a long tradition of hosting UN and other international organizations such as the World Health Organization, the World Meteorological Organization, and the World Trade Organization. Because much of the IRGC's work addressed transboundary risk governance, it benefited considerably from being based in Switzerland. In addition, Switzerland has a strong history of expertise in risk‐related work in government, industry, and academia, upon which IRGC was able to build.

Charles Kleiber, then head of the SERI of the Swiss Federal Government, was a driving force in creating IRGC as a free‐standing nonprofit Foundation.

IRGC acted as a multistakeholder neutral convening platform for policymakers, scientists, and the private sector to discuss and perform analysis on the challenges of risk governance. IRGC's decision‐making was led by a Foundation Board, initially chaired by José Mariano Gago, Minister of Science and Technology of Portugal. Wolfgang Kröger of Eidgenössische Technische Hochschule Zürich (ETHZ) and the Paul Scherrer Institute (PSI) was IRGC's Founding Rector. A Scientific and Technical Council (S&TC), chaired by Granger Morgan of Carnegie Mellon University, was responsible for drawing up IRGC's work program and overseeing IRGC's substantive research and policy activities. Christopher Bunting was appointed to be the Secretary General of the organization, followed later by Marie‐Valentine Florin.

Other key players in the Foundation's early days included Bruno Porro and Christian Mumenthaler of Swiss Re, Pierre Béroux of EDF, Manuel Heitor of the Government of Portugal, Donald J. Johnston, who was Secretary General of the OECD, and nuclear regulatory expert Manning Muntzing.

From 2003 to 2012, support for the effort came from SERI and the Swiss Agency for Development and Cooperation, as well as from Swiss Re, Électricité de France (EDF), ATEL Holding AG (now part of ALPIQ Holding AG), Oliver Wyman, E.ON AG (now E.ON SE), and financial or in‐kind support from the Governments of Austria, the US, China, and South Korea.

IRGC's initial program was established by the Board and S&TC in 2004 and consisted of three main bodies of work, of which one, focused on the core concepts of risk governance, was to remain central to IRGC's work throughout its lifetime.^3^

**FIGURE 1:**
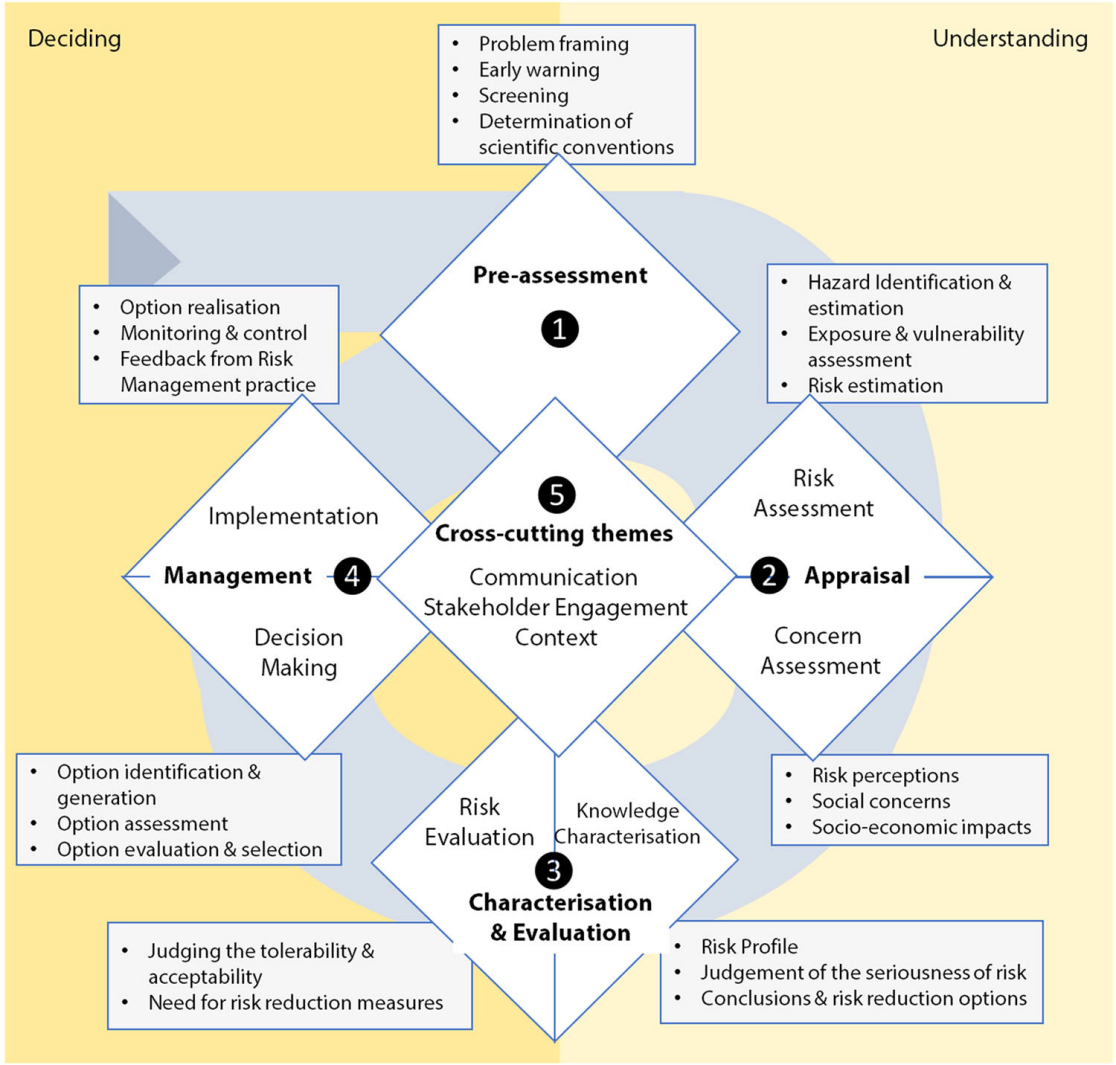
Diagrammatic summary of the IRGC Risk Governance Framework. A detailed explanation of the framework and its use can be found online at: https://irgc.org/wp‐content/uploads/2018/09/IRGC.‐2017.‐An‐introduction‐to‐the‐IRGC‐Risk‐Governance‐Framework.‐Revised‐version.pdf.

Ortwin Renn of the University of Stuttgart^4^ led the development of IRGC's Risk Governance Framework (Figure 1) that established the conceptual foundation for much of the IRGC's work in the years that followed. The Risk Governance Framework is based on a broad analysis of evidence‐based approaches to risk assessment, management, and communication. Its purpose is to provide policymakers, regulators, and risk managers with methodological orientation and empirical evidence to cope with risk governance challenges. It is a generic and adaptable framework that can be tailored to address major risks, including systemic risks. An initial description of the framework was published in a 2005 IRGC White Paper titled *Risk Governance—Towards an Integrative Approach*. A summary version was published in 2017, including some practical experiences of the use of the framework in risk governance agencies (IRGC 2017a). The implications of the risk governance framework for stakeholder involvement were also summarized in a web‐based guidance document and an IRGC report (IRGC 2020a).

Building on this work and on feedback from practical applications, IRGC's 2019 report on *Risk Governance Deficits: An analysis and illustration of the most common deficits in risk governance* (IRGC 2019)^5^ focused on the sources of governance deficits and their constructive assessment and management. Further to this, IRGC produced a series of publications to address emerging risks, in particular: *The Emergence of Risks: Contributing Factors* (IRGC 2010a) and *Emerging Risk Governance Guidelines* (IRGC 2015a). These publications expand and build on the main risk governance framework to address issues specific to emerging risks.

**FIGURE 2:**
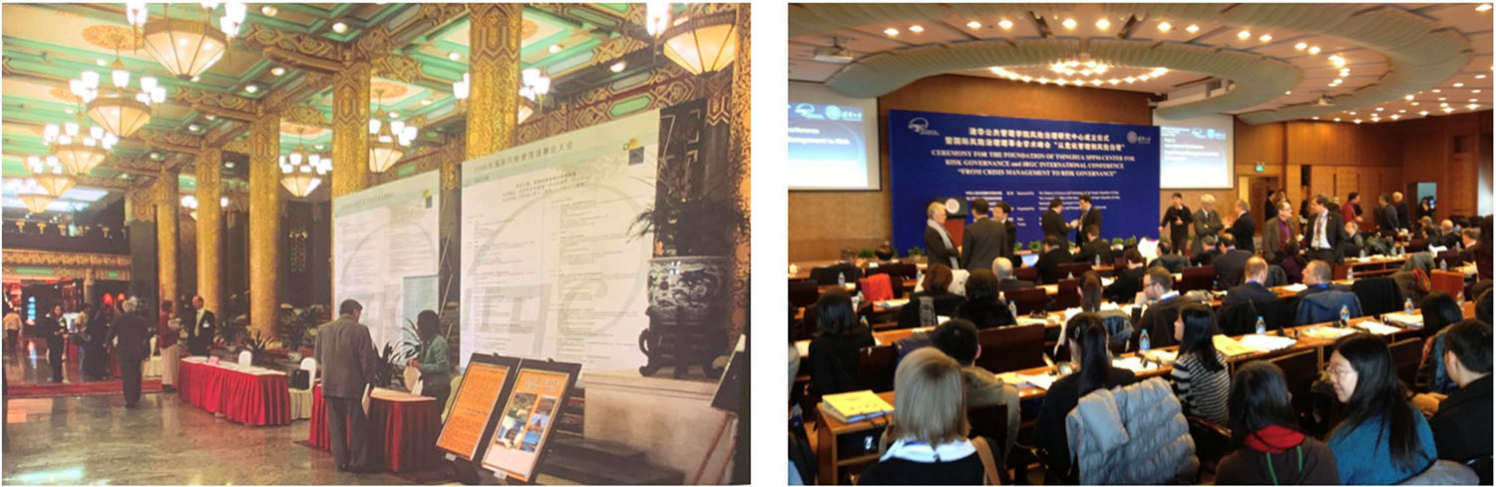
Views of the two international conferences IRGC ran in Beijing, China in 2005 (left) and 2013 (right).

**FIGURE 3:**
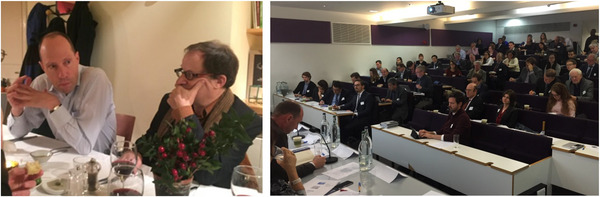
Views of the 2016 London Conference. Image at the left shows conference organizer Arthur Petersen of UCL, with Gérard Escher of EPFL.

In addition to conducting studies on a range of important and emerging risk‐related issues, IRGC organized and ran many workshops as well as international conferences in Beijing in 2003 and 2013 (Figure 2) and London in 2016 (Figure 3). The Beijing conferences were hosted by LIU Yanhua, China's Vice Minister for Science and Technology. S&TC member XUE Lan of Tsinghua University played a major role in organizing and supporting these and other activities in China and served for years as a member of the organization's Scientific and Technical Council. Arthur Petersen hosted the London conference that was co‐organized with the Department of Science, Technology, Engineering and Public Policy at University College London (UCL STEaPP).

A list of the reports that IRGC published between 2003 and 2012 is provided in Table 1. Copies of these reports have been archived and can be accessed at https://irgc.org/publications/.

**TABLE 1:** Reports published by IRGC between 2003 and 2012 while the organization was operating as a freestanding Swiss foundation.

**Part 1. Publications on specific issues** **Air Quality** The Linkages Between Air Quality and Climate Policies: Governance Deficits and Challenges (Concept Note, 2008) **Bioenergy** Risk Governance Guidelines for Bioenergy Policies (Executive Summary, 2008) Risk Governance Guidelines for Bioenergy Policies (Policy Brief, 2008) Governing the Risks and Opportunities of Bioenergy (Concept Note, 2007) **Carbon Capture and Storage** Power Plant CO2 Capture Technologies (Concept Note, 2009) Regulation of Carbon Capture and Storage (Policy Brief, 2008) **Critical Infrastructure** Risk Governance of Maritime Global Critical Infrastructure (Report, 2011) Managing and Reducing Social Vulnerabilities from Coupled Critical Infrastructures (Policy Brief, 2007) Managing and Reducing Social Vulnerabilities from Coupled Critical Infrastructures (White Paper, 2006) **Climate Engineering/Solar Radiation Management** Cooling the Earth Through Solar Radiation Management: The Need for Research and an Approach to its Governance (Opinion Piece, 2010) **Nanotechnology** Appropriate Risk Governance Strategies for Nanotechnology Applications in Food and Cosmetics (Policy Brief, 2009) Risk Governance of Nanotechnology Applications in Food and Cosmetics (Report, 2008) Nanotechnology Risk Governance (Policy Brief, 2007) Nanotechnology Risk Governance (White Paper, 2006) **Pollination Services** Risk Governance of Pollination Services (Concept Note, 2009) **Social Media and Crisis Communication** Addressing the Challenges of Using Social Media to Improve Crisis Communication and Management (Concept Note, 2012) **Synthetic Biology** Guidelines for the Appropriate Risk Governance of Synthetic Biology (Policy Brief, 2010) Risk Governance of Synthetic Biology (revised Concept Note, 2009) **Part 2. Core concepts of risk governance** **Risk Governance Framework** Global Risk Governance—Concept and Practice Using the IRGC Framework (2008) Edited by Ortwin Renn and Katherine Walker, IRGC Book series 1 published by Springer Risk Governance—Towards an Integrative Approach (White Paper, 2005) **Risk Governance Deficits** Risk Governance Deficits (Policy Brief, 2010) Risk Governance Deficits (Report, 2009) Risk Governance Deficits (Concept Note, 2008) **Governance of Emerging Risks** Improving the Management of Emerging Risks (Concept Note, 2011) The Emergence of Risks: Contributing Factors (Report, 2010) The Emergence of Risks: Contributing Factors (Executive Summary, 2010) Emerging Risks: Sources, Drivers and Governance Issues (revised Concept Note, 2010)

*Note*: Part 1 lists publications on specific issues, and Part 2 lists publications on core concepts of risk governance. Hyperlinks are included in the online version of this paper.

After IRGC had operated as a free‐standing foundation for almost a decade, the Swiss State Secretariat for Education and Research performed an extensive review of IRGC's added value and proposed to have IRGC hosted and supported by an academic institution from the ETH domain^6^ so that IRGC might work more closely with, and provide more benefit to, Swiss research and education. Consideration was given to moving to either ETHZ (Zurich) or EPFL (Lausanne). Philippe Gillet, who was then Vice‐President of Academic Affairs at EPFL, indicated that the University would create an International Risk Governance Center, so, in June of 2012, the IRGC secretariat moved its offices from Geneva to the EPFL campus in Lausanne. By this time, Marie‐Valentine Florin had become Managing Director with responsibility for running the organization. EPFL's Gérard Escher facilitated many of the new arrangements. From 2012, most of the funding came from EPFL.

One of IRGC's greatest strengths was the formal and informal international network of experts that it developed and maintained.^7^ Many members of this network served as members of the Scientific and Technical Council. Others simply developed long‐standing associations with IRGC and assumed leadership roles in a number of its studies and programs.

Table 2 summarizes the publications from IRGC during the period from 2012 to 2023. Again, copies of these reports have been archived and can be accessed at https://irgc.org/publications/ or https://irgc.epfl.ch.

**TABLE 2:** Reports published by IRGC between 2012 and 2023 while the organization was operating within the ETH Domain at EPFL in Lausanne.

**Part 1. Publications on specific issues** **Autonomous Cars** Risk and Opportunities Governance of Autonomous Cars (Background Paper, 2016) **Climate Engineering** International Governance Issues on Climate Engineering (Report, 2020) **Critical Infrastructure** Critical Infrastructure Resilience—Lessons from Insurance (Policy Brief, 2019) **Digitalization** Governing, Opportunities and Risks of Digital Currencies (Workshop Highlights, 2022) Governance of and by Digital Technology—Conference Proceedings (Conference Proceedings, 2020) Forged Authenticity: Governing Deepfake Risks (Policy brief, Slide presentation, 2019) The Governance of Decision‐Making Algorithms (Workshop report, Presentation slides, 2018) Governing Risks and Benefits of Distributed Ledger Technology Applications (Workshop Highlights, 2017) Governing Cybersecurity Risks and Benefits of the Internet of Things: Connected Medical and Health Devices and Connected Vehicles (Workshop report, Workshop Highlights, 2017) Cyber Security Risk Governance (Workshop Report, 2016) Public Cybersecurity and Rationalizing Information Sharing (Opinion Piece, 2016) Comparing Methods for Terrorism Risk Assessment with Methods in Cyber Security (Workshop Report, 2015) **Energy Efficiency** The Rebound Effect: Implications of Consumer Behavior for Robust Energy Policies (Report, 2013) **Energy Transitions** Demand‐Side Flexibility for Energy Transitions: Policy Recommendations for Developing Demand Response (Policy Brief, 2016) Demand‐Side Flexibility for Energy Transitions: Ensuring the Competitive Development of Demand Response Options (Report, 2016) Assessment of Future Energy Demand: A Methodological Review Providing Guidance to Developers and Users of Energy Models and Scenarios (Concept Note, 2015) Risk Governance and the Low‐Carbon Transition (Policy Brief, 2021) **Nuclear Energy** Preserving the Nuclear Option: Overcoming the Institutional Challenges Facing Small Modular Reactors (Opinion Piece, 2015) **Precision Medicine** The Economics of Precision Medicine (Workshop Report, 2018) Governance of Trust in Precision Medicine (Workshop Report, 2018) Roadmap for Precision Medicine (Policy Brief, 2017) Collection, Access and Use of Human Genetic Information for Precision Medicines: Risk Governance Considerations (Workshop Report, 2016) (pdf) **Social Media and Crisis Communication** Addressing the Challenges of Using Social Media to Improve Crisis Communication and Management (Concept Note, 2012) **Space Debris** Policy Options to Address Collision Risk from Space Debris (Policy brief/Presentation slides, 2021) Collision Risk from Space Debris: Current Status, Challenges and Response Strategies (Report / Presentation slides, 2021) **Synthetic Biology** Emerging Threats of Synthetic Biology and Biotechnology (Book, 2019) Security for Emerging Synthetic Biology and Biotechnology Threats (Workshop Proceedings, 2019) **Unconventional Gas Development (shale gas)** Risk Governance Guidelines for Unconventional Gas Development (Policy Brief, 2014) Risk Governance Guidelines for Unconventional Gas Development (Report, 2014)
**Part 2. Core concepts of risk governance** **Risk Governance Framework** Introduction to the IRGC Risk Governance Framework—A Revised Version (2017) Stakeholder Involvement: Involving Stakeholders in the Risk Governance Process (Guidebook, 2020) **Governance of Emerging Risks** IRGC Guidelines for Emerging Risk Governance (Report, 2015) Appendix to the IRGC Guidelines for Emerging Risk Governance (Report Appendix, 2015) Public Sector Governance of Emerging Risks (Concept Note, 2013) Public Sector Governance of Emerging Risks. Hallmarks and Drivers (Workshop Report, 2013) **Governance of Systemic Risks** IRGC Guidelines for the Governance of Systemic Risks (Report/Slide presentation, 2018) **Resilience** Resource Guide on Resilience, Volume 2 (Compilation of authored pieces, 2018) Resource Guide on Resilience, Volume 1 (Compilation of authored pieces, 2016) **Risk Regulation** Transatlantic Patterns of Risk Regulation (Report, 2017) Planning Adaptive Risk Regulation (Conference report, 2016) Improving Risk Regulation (Report, 2015) **Slow‐Developing Catastrophic** **Risks** Governance of Slow‐Developing Catastrophic Risks: Fostering Complex Adaptive System and Resilience Thinking (Report, 2015) **Ensuring the Environmental Sustainability of Emerging Technology Outcomes** Report 3, Guidance to Various Actors (Report, 2023) Report 2, Learning and Applying IRGC's Finding to Various Emerging Technologies (Edited Volume, 2023) Report 1, Setting the Scene: Concerns, Cross‐Sectoral Aspect and Response Strategies (Workshop Report, 2022)

*Note*: Part 1 lists publications on specific issues, and Part 2 lists publications on core concepts of risk governance. Hyperlinks are included in the online version of this paper.

In its new setting at EPFL, IRGC participated in EPFL's broad research and education mission, while continuing to run its own activities. Those activities prioritized issues in which emerging technologies or their applications could create risks or raise challenges in the future. The intention was to direct attention to technology‐related matters that look promising at first sight, but whose developments ought to be carefully overseen so that while they unlock economic or social opportunities, they do not cause unfair or unsustainable risks. In its later years, IRGC's dual nature as an academic institution that acted as a think tank, as well as its fully international nature that targeted a broad audience, created difficulties in finding additional financial support. After exploring the possibility of merging with several other Swiss entities, a decision was made to close IRGC and transfer its assets to the Laboratory for Energy Systems Analysis (LEA) of the PSI (one of the four research institutes within the ETH domain). EPFL has archived the IRGC website and publications. LEA is using remaining IRGC funds to conduct a study on risks related to the Energy Transition, to maintain the public access to IRGC's publications, and to organize IRGC's legacy for further dissemination and use.^8^ In early October 2024, it hosted a final “Reflection Workshop” near Zurich that involved a number of former IRGC participants and others, including the authors of this paper and members of PSI staff.

## 2 Six Examples of IRGC Contributions

In this section, we briefly recount six examples of some of IRGC's focal activities. Some of the many other topics on which IRGC made important contributions are nanotechnology, bioenergy, pollination services, the linkages between air quality and climate policies, aftificial intelligence (AI) decision‐making algorithms, deepfakes, the management of space debris, and ensuring the environmental sustainability of emerging technology outcomes.^9^

Example 1 **Solar Radiation Management (SRM)**

As the world continues to fall short of achieving a level of decarbonization sufficient to slow and ultimately reverse climate change, discussion of the topic of SRM (increasing planetary albedo to slow global warming) has become increasingly common. In its role of anticipating emergent technologies and associated risks, IRGC was one of the first organizations to identify and address this important topic.

Aware of a forthcoming recommendation that the foreign policy community should be better informed about this issue (Victor et al. 2009), the IRGC, together with the government of Portugal, Carnegie Mellon University, and the University of Calgary, sponsored a workshop in Lisbon at the Gulbenkian Foundation with participants from North America, the EU, China, Russia, and India.

Following the workshop, in 2010, IRGC published a report titled “Cooling the Earth Through Solar Radiation Management: The need for research and an approach to its governance” (IRGC 2010b).

The report argued that SRM should not be viewed as an alternative to reducing the emission of greenhouse gases but rather as something that might be needed in a global emergency. It laid out two reasons why research is needed on SRM:

ʻ1. There is a growing chance that some part of the world will find itself pushed past a critical point where, for example, patterns of rainfall have shifted so much that agriculture in the region can no longer feed the people, and heat waves kill thousands. Because this shift is the result of rising global temperatures, such a region might be tempted to unilaterally start doing SRM to solve its problem. If this situation arises, and no research has been done on SRM, the rest of the world could not respond in an informed way.

2. With luck, the major effects of climate change will continue to occur slowly, over periods of decades. However, if the world is unlucky and a serious change occurs very rapidly, the countries of the world might need to consider collectively doing SRM. If this situation arises, and no research has been done, SRM would involve a hopeful assumption that the uncertain benefits would outweigh the uncertain and perhaps unknown costs.ʼ^10^

The report went on to summarize what was known at the time about potential risks from engaging in SRM and then explored how research could be safely done in the atmosphere without creating any significant risks, defining a multidimensional “allowed zone.”

**FIGURE 4:**
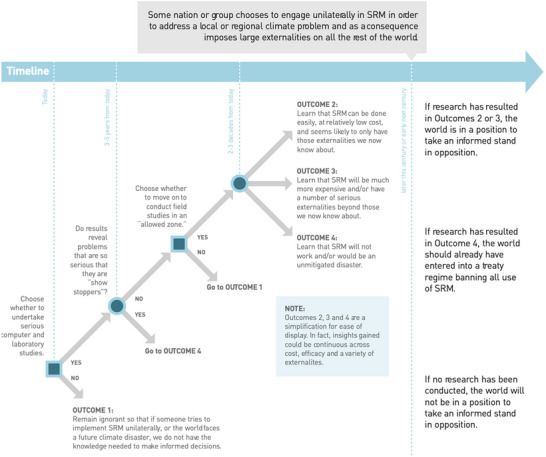
One of a series of diagrams contained in the IRGC report illustrating possible future outcomes if the world did or did not engage in serious research on SRM. This particular diagram shows the situation that would result in the future if some major state decided to deploy SRM unilaterally in order to address a local original impact from climate change.

The report included a series of diagrams that showed the situation the world might face if research had or had not been undertaken at a time when a nation or private actor chose to unilaterally engage in SRM. An example is shown in Figure 4.

Because the topic of SRM was (and is) highly controversial, the IRGC Board was not comfortable releasing this report as an official product of the organization, and so initiated a strategy of signed authored reports, a strategy that was subsequently adopted for dealing with a few other controversial topics.

Building on the background, a decade later, in 2020, IRGC and the Carnegie Council on Ethics and International Affairs ran a joint workshop on SRM Governance. This, in turn, led to an invitation to IRGC from the Swiss Federal Office for the Environment (FOEN), International Affairs Division, to review the topic and produce a report “International Governance Issues on Climate Engineering.” The four chapters, authored by experts in the field, draw together information and recommendations relevant to international policymaking in this area of growing importance to mitigating the effects of climate change.  The report argued that “Policy decisions must strive to be based on evidence and a shared, robust understanding of the potential opportunities and risks, across disciplinary and applied perspectives” (IRGC 2020b).

Example 2 **Small Modular Reactors (SMRs)**

As the world struggles to decarbonize the energy system, SMRs, which some argue might be made affordable through factory manufacturing (Lloyd et al. 2021; Abdulla et al. 2013), are often advanced as a possible part of a broader portfolio of low‐carbon energy sources.

While it is unclear if such reactors will become affordable and make a significant contribution to decarbonization in the next several decades (Morgan et al. 2018), if they ever do become widely deployed, a related concern is whether their wide deployment would raise risks of fuel diversion and contribute to nuclear proliferation.

**FIGURE 5:**
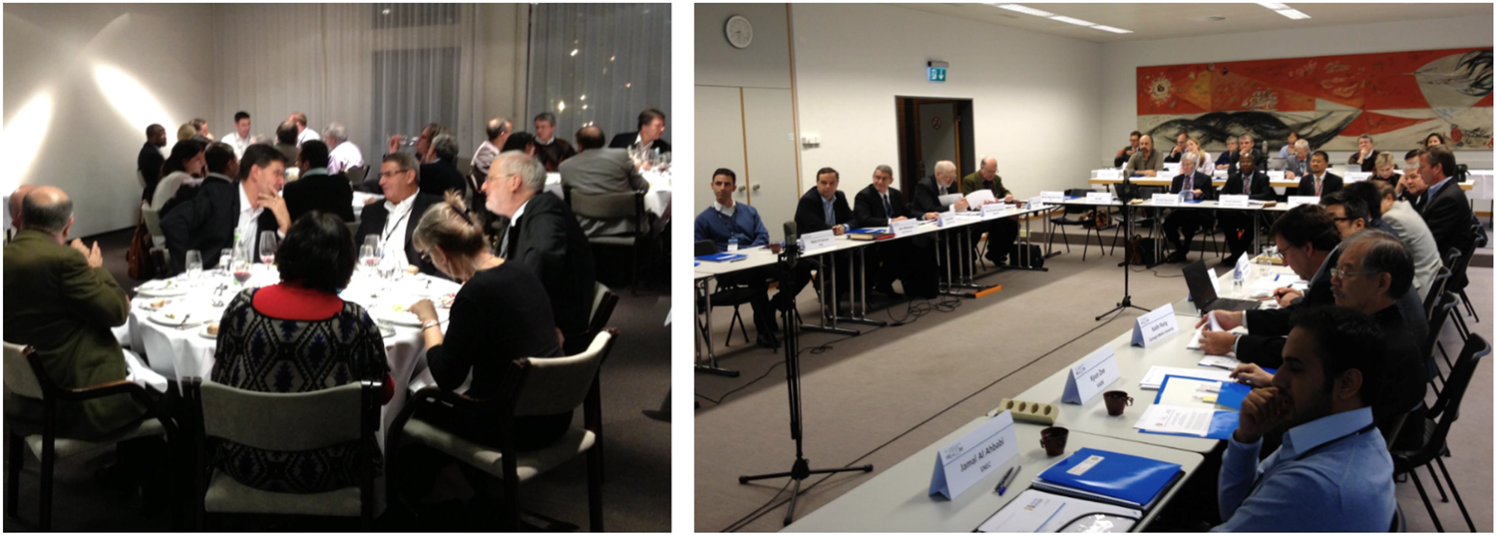
Two views of the workshop on small modular reactors held at the Paul Scherer Institute (PSI) in November 2013.

To assess those issues, in November of 2013, IRGC, together with investigators in the Department of Engineering and Public Policy at Carnegie Mellon University and the PSI, convened a workshop at PSI in Villigen, Switzerland which brought together representatives from 40 SMR vendors, nuclear utilities, regulatory bodies, universities, and national laboratories from around the world (Figure 5). The workshop format was unusual in that sessions alternated back‐and‐forth between formal presentation and the use of very detailed workbooks in which participants recorded their views about technical issues and proliferation potential associated with six different SMR reactor designs (2 light water; 2 liquid metal; 2 gas cooled).

**FIGURE 6:**
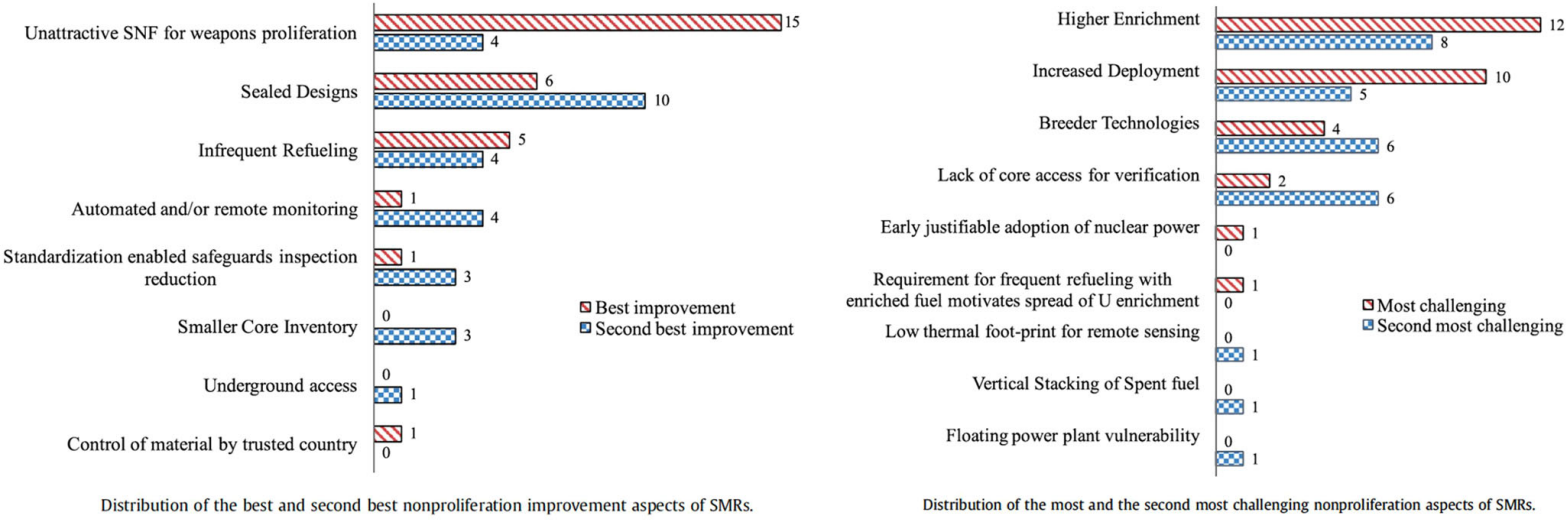
Summary of expert assessments about proliferation risks associated with small modular reactors (SMRs).

Results from the workshop were reported in a paper in *Progress in Nuclear Energy* (Prasad et al. 2015). Figure 6 summarizes some of the experts’ assessments.

A more general discussion of issues related to nuclear power across the developing world was also published in *Issues in Science & Technology* (Abdulla and Morgan 2015).

Without taking a position on whether it supported the expanded use of nuclear power as part of a decarbonization strategy,^11^ IRGC commissioned Ahmed Abdulla to prepare a signed opinion piece on SMRs, which the organization published as a report titled *Preserving the Nuclear Option: Overcoming the institutional challenges facing small modular reactors* (IRGC, 2015b). The report noted that:

“… overcoming nuclear power's challenges requires changes in the existing construction, deployment, and institutional paradigms that govern the technology. Such changes may be catalyzed by the development and deployment of small modular nuclear reactors (SMRs), which would complement large light water reactors (LWRs), or perhaps be used by emerging nuclear energy states to gain experience with nuclear power operation, before moving on to larger units. SMRs can produce electricity, and can also provide services such as desalination or district heating. Small nuclear reactors have the potential to improve performance in nuclear power generation by enhancing their performance across several areas, including safety of reactor operations, waste management, proliferation, and high economic cost. Perhaps the most promising SMRs are those that could be fabricated and fueled in an internationally supervised factory, shipped to a site where they operate without refueling, and are then removed upon end‐of‐life to an internationally supervised waste processing facility. The main feature of SMRs is their smaller size, which guarantees greater affordability in terms of the total upfront capital that needs to be made available for each project. Economic competitiveness can be improved through mass fabrication on a factory assembly line, allowing modularity. Most designs rely on passive safety systems to manage the consequences of an accident. Finally, waste recycling concerns can be addressed with long core‐lives: some novel SMRs are able to operate for up to thirty‐two years without refueling and, once the fuel is exhausted, the reactor module is extracted from its vault in one piece and shipped to a secure facility for processing.”

However, it is important to note that many obstacles would have to be overcome for SMRs to achieve mass deployment. First, the commercial nuclear industry has very little experience with untested technical paradigms such as underground or sea‐based reactors. Second, there are many institutional challenges, and strong political backing would be needed to overcome many of them. Current international treaties are not an impediment to the development and mass deployment of SMRs, but many national regulatory regimes do impose large barriers on SMR development and deployment. As far as the global liability regime is concerned, more than half of the world's nuclear facilities are not covered by any liability regime currently in effect.

SMRs face institutional challenges. In the case of emerging nuclear energy countries, there is little institutional support—on a transnational or even international level—for states that do not have a framework in place to purchase, build, and run nuclear power plants on their own. They would benefit from help with issues that involve security, human capital development, accident response, or managing complex projects. More research on the following fronts would help SMR development:

- Comparative risk assessment of alternative SMR deployment options and technologies. Bilateral and multilateral agreements on enhanced nuclear safety and security.
- Definition of the minimum emergency infrastructure that is needed for safe and secure operation of SMR plants.
- A global liability regime that ensures all reactors are covered by currently existing programs, perhaps coupled with the development of viable alternatives or supplementary regimes on the regional level.

Example 3 **Synthetic Biology**

Synthetic biologists apply engineering principles to develop new biological parts, devices, and systems and redesign existing natural systems (Oye and Wellhausen 2009). From 2008 to 2019, IRGC sought to improve the terms of tradeoffs across benefits and risks of synthetic biology (Oye 2012). During this period, the evolution of relevant technologies and uncertainty over the likelihood of benign and malign applications complicated risk governance (Steinbruner and Okutani 2004). IRGC's work in this domain^12^ took account of the unusually rapid changes in underlying technologies with complex implications for the organization of communities of synthetic biologists.

Synthetic biologists are initially divided into two groups with distinct applications and risks:

- The NSF Synthetic Biology Engineering Research Center and the International Genetically Engineered Machine competition focused on modular design, with emphasis on creating libraries of interchangeable genomic parts with standardized couplings and then combining those parts into useful biological systems. Modularity lowered the skill thresholds required to do genetic engineering. Risk governance issues centered on the diffusion of synthetic biology from universities to high schools, from corporate labs to garages, and from advanced industrial countries to the developing world.
- The J. Craig Venter Institute focused on prospecting for genetic sequences in nature, on editing genetic sequences to enhance functionality, and on creating artificial life forms stripped of inessential genetic elements. Risk governance issues centered on safety, security, and environmental implications associated with the creation of novel life forms, from incremental enhancements to artificial life.

Technical advances soon blurred the distinction between these groups. CRISPR and base editing increased the power and decreased the difficulty of gene editing, while Gibson Assembly facilitated joining genetic elements without standardized couplings. These changes accelerated the diffusion of synthetic biology without modularization. Finally, the emergence of digitized data sets integrating genomic information with clinical, agricultural, and environmental data and the use of AI to analyze data and provide a rigorous basis for directing gene editing have greatly enhanced the power and efficiency of genetic engineering while introducing risks associated with maintaining the privacy of individuals in data sets.

International differences in societal and regulatory context are also a basic feature of the synthetic biology risk governance landscape. US acceptance of agricultural applications of genetic engineering contrasts with EU concerns over the environmental effects of recombinant genetically engineered crops and the safety of genetically modified foods. But the US and EU do not differ significantly in the governance of risks of biomedical applications and the applications of contained synthetic biological materials. Furthermore, the EU is now differentiating between the use of editing of genetic elements as distinct from trans‐species recombination of genetic elements. In short, stark differences between US and EU approaches to the regulation of genetic engineering are gradually diminishing.

The first phase of IRGC's work on synthetic biology reckoned with significant uncertainty by invoking the need for adaptive approaches to risk governance. This work took place as IRGC advisory group members were working with the European Medicines Agency on the development of adaptive approaches to licensing of pharmaceuticals and borrowed from that work. In synthetic biology, pervasive uncertainty over the feasibility of potential beneficial materials production, agricultural and medical applications, over the intensity of societal reactions to acceptance of risks and blocking of benefits, and over the effectiveness of voluntary guidance and mandatory regulatory responses ensures that initial takes on risk governance will be based on incorrect assumptions. IRGC's work in this space emphasized the need for establishing baselines, gathering updated information, and establishing procedures for modification of initial policies in light of evolving information.

The second phase of IRGC's work on synthetic biology built on the foundation it had developed on adaptive risk governance, with added emphasis on complications introduced by information hazards. For example, in 2019, IRGC organized a workshop under the auspices of NATO's Science for Peace and Security Program^13^ to consider the security implications of synthetic biology. In this realm, information on potentially malevolent applications of synthetic biology and associated methods and tools is necessary in order to identify and address security and safety concerns. Biosecurity officials, biosafety officers, and researchers need to know what is dangerous to manage risks. At the same time, should that information fall into the hands of malevolent or incompetent actors, biosecurity and biosafety risks could be intensified. The NATO workshop and subsequent IRGC publications addressed this difficult problem.

Example 4 **Autonomous Vehicles (AVs)**

It has been clear from the outset of the development of technologies that enable personal vehicles to drive fully autonomously, that a number of uncertainties will need to be resolved (including issues of standards and interoperability, safety, public acceptance, cyber security, and liability in case of accident). IRGC addressed this topic in January 2016 as part of its London Conference on “Planning Adaptive Risk Regulation” (IRGC 2016)^14^ and then in June 2016 in a dedicated workshop in Zurich.

The report on the London conference notes that the safety issue is a prerequisite for addressing both the opportunities and challenges. It asks “how safe should autonomous vehicles (AVs) be before they are allowed on the roads, and how do we prove they are safe? While humans can make mistakes, there is a cultural aversion to ‘letting machines make mistakes’. Some will insist that for introducing AVs, anything short of totally eliminating risk is a safety compromise. However, waiting for autonomous vehicles to be perfect itself raises safety concerns, because it would mean the needless perpetuation of the well‐documented risks posed by human drivers” and suggests that “AVs might optimally be introduced when they are just somewhat safer than human drivers (perhaps for use by the least safe human drivers)—or arguably even when the AVs are not yet quite as safe as (safer) human drivers, because this earlier introduction of still‐imperfect AVs can enable faster learning to improve AVs so that AVs more rapidly outperform human drivers and thereby reduce overall driving risks more steeply” (IRGC 2016).

One of the workshop's key takeaways was that “adaptive pathways whereby regulators, industry and society collaboratively learn how to manage risks and benefits from the technology are desirable to manage fears, risks and incidents, and progressively develop regulations. It might be preferable to make exceptions to current regulations, rather than trying to fix things with new regulations and laws before sufficient experience has been collected through real cases, small steps, failures, learning‐by‐doing, and collaboration.”^15^

In 2020, IRGC further explored the topic, comparing and contrasting advances in the development of AVs and in connected medical devices, focusing on cybersecurity risks. Because AVs are digitally connected, one should make sure that cybersecurity cannot be compromised.^16^

Example 5 **Resilience and Systemic Risk**

*Resilience*: IRGC's work on resilience represented an early effort to develop systematic frameworks for understanding and implementing resilience approaches within risk governance. IRGC's engagement with resilience concepts began with the publication in 2003 of the Risk Governance Framework, which includes resilience building among various possible risk management strategies.

Foundational publications like the US National Academy “Disaster Resilience: A National Imperative” (NASEM 2012) and the US Presidential Policy Directive 21 on Critical Infrastructure Security and Resilience (EOP 2013) signaled a growing interest in resilience across academic and policy spheres. These developments highlighted resilience management as a complement to risk management for addressing complex threats to critical infrastructure and social systems.

IRGC's first major contribution to resilience scholarship^17^ was the 2016 “Resource Guide on Resilience Volume 1,” which compiled perspectives from international experts to examine theoretical foundations and practical applications of resilience concepts. The guide addressed resilience measurement, implementation challenges, and integration with existing risk assessment frameworks. Contributors analyzed resilience through multiple disciplinary lenses, including engineering systems, ecology, psychology, and organizational management. The guide established key distinctions between resilience and traditional risk management approaches, positioning resilience as a framework for addressing high uncertainty and complexity in interconnected systems, especially when managers are unable to fully prepare for potentially large‐scale risk consequences. Volume 1 emphasized resilience as the underlying capacity for systems to maintain core functions through adaptation, and even transformation to some extent, when faced with both anticipated and unexpected disruptions.

Building on this foundation, IRGC published “Resource Guide on Resilience Volume 2” in 2018, which expanded the theoretical scope while providing concrete guidance for practitioners. Volume 2 examined emerging resilience applications in areas such as critical infrastructure, cybersecurity, climate adaptation, and supply chain management. The guide highlighted methodological approaches for resilience assessment and measurement, including network analysis, agent‐based modeling, and resilience indicators. Case studies demonstrated how organizations implemented resilience frameworks to enhance system stability and adaptive capacity. Volume 2 emphasized the role of governance structures, institutional arrangements, and stakeholder engagement in building system‐wide resilience.

IRGC's contributions to resilience scholarship and practice include several defining elements. First, IRGC positioned resilience within the broader risk governance framework, emphasizing complementarity between traditional risk management and resilience approaches, while denoting the unique challenges and opportunities for practitioners seeking a resilience‐centered approach grounded in systemic recovery and adaptation. This integration helped organizations incorporate resilience thinking into existing risk assessment and management processes. Second, IRGC's work highlighted the multiscale nature of resilience, examining interactions between technical, organizational, and social dimensions of resilient systems. This systems perspective informed approaches for building resilience across interconnected infrastructure networks and institutions. Third, IRGC emphasized the dynamic nature of resilience, focusing on recovery, adaptation, and transformation as essential capabilities for maintaining system function through changing conditions. Resilience is a key capability for handling complexity and uncertainty and navigating transitions and transformations in complex adaptive systems facing systemic risks, thus for addressing complex global challenges such as climate change, degradation of ecosystem services, or information security.

*Systemic risk*: IRGC's 2018 “Guidelines for the Governance of Systemic Risks” (IRGC 2018)^18^ represented a synthesis of resilience concepts with systemic risk governance and intended to guide organizations in understanding complex system dynamics and reflecting on their position within these dynamics. The guidelines help actors in a system to either prevent the shift of the system within which the organization operates to an undesirable regime, or trigger and facilitate the transition of the system to a preferable regime, considering changes in underlying context conditions or proximity to a tipping point that may trigger an undesirable regime shift.

**FIGURE 7:**
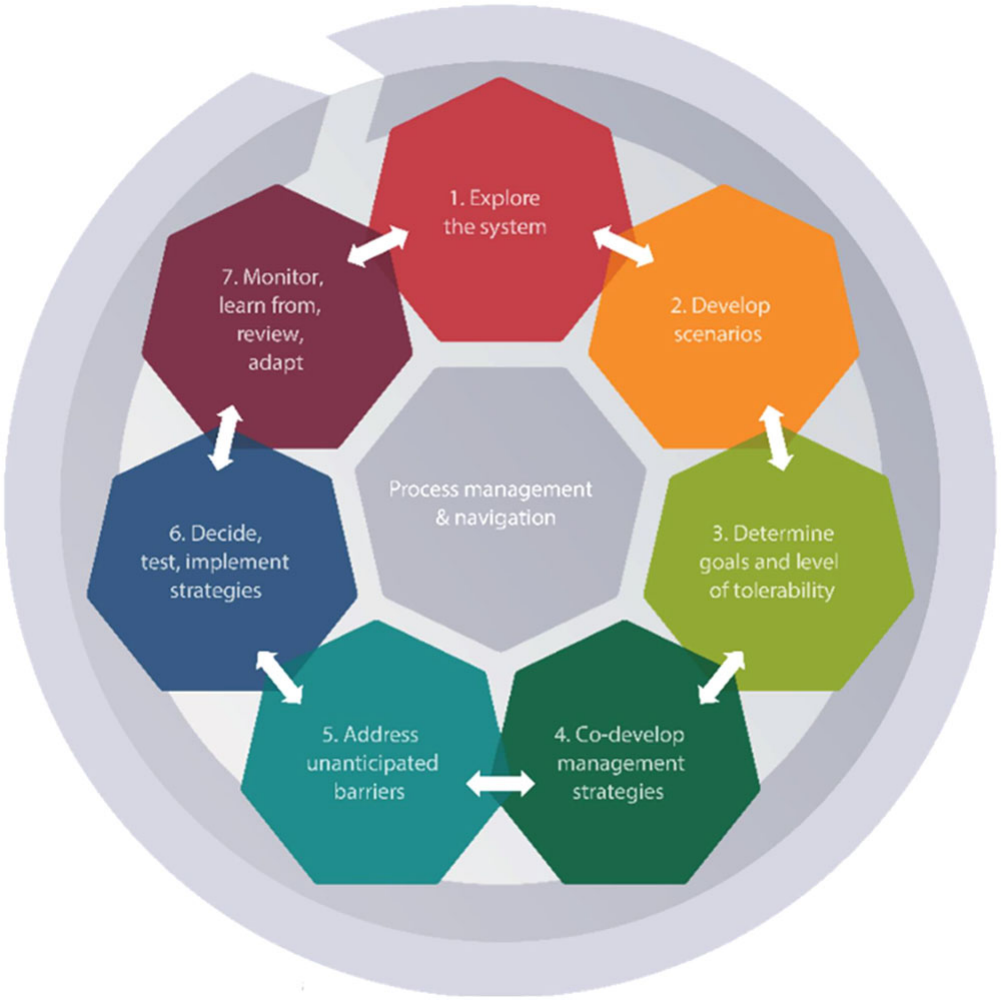
Elements of the IRGC Systemic Risk Governance Guidelines. Resilience assessment is included under Step 1, and building resilience as one of the possible risk management strategies is included in Step 4.

The guidelines outlined a structured process for organizations to understand their position within complex adaptive systems and develop various strategies for addressing systemic risks, among which are resilience‐based approaches(Figure 7). With large organizations as an intended audience, the guidelines focus on interconnectedness and the potential for catastrophic failures that characterize systemic risks. They emphasize three core strategic elements: the benefits of enhancing system self‐organization capabilities; the need to implement proactive interventions through prevention, mitigation, adaptation, and transformation; and the necessity to prepare for disruptions through scenario planning, response capacity building, and resilience building.

The COVID‐19 pandemic in 2020 demonstrated the relevance of IRGC's framework and guidelines for understanding and responding to global systemic crises. The pandemic highlighted interconnections between public health, economic, and social systems while revealing vulnerabilities in critical infrastructure and supply chains. Organizations and governments drew on resilience concepts to develop adaptive responses and build preparedness for future disruptions. IRGC's prior work provided theoretical foundations and practical guidance for implementing resilience approaches during the crisis.^19^

Example 6 **International Comparison of Risk Governance**

Disputes and controversies can arise from differences across jurisdictions in risk regulations and governance approaches. These differences may become barriers to international trade, and may yield litigation, retaliatory trade measures, negotiation, and efforts at international regulatory cooperation. At the same time, variation across jurisdictions in risk regulations and governance approaches can also be a source of useful learning, furnishing comparative empirical experience with policies that can help observers better understand policy options and associated outcomes, and thereby help improve risk governance.

IRGC undertook a study of regulatory differences between the United States and Europe, producing a public report and a briefing to the European Parliament (IRGC 2017b). The IRGC study compared US and EU risk regulation overall, and in four key sectors: food, automobiles, pharmaceuticals, and chemicals. The report observed that transatlantic differences in risk governance are complex and multifaceted, exhibiting selective application of stringency and precaution regarding particular risks in both the US and EU, rather than distinct US and EU approaches:

“[O]ne viewpoint is that European regulatory standards have become more protective – more stringent and precautionary – than US regulatory standards, so that mutual recognition of current standards, or convergence on a new harmonized standard, might weaken European standards (although ‘harmonizing up’ to higher standards is also possible). But the real pattern of actual regulation is more complex. Stringent policies have been pursued on both sides of the Atlantic, with frequent parity and occasional particular variation in both directions (sometimes greater European stringency, sometimes greater US stringency). Impact assessment and cost‐benefit analysis of regulation have also been employed on both sides. A key problem in claims of one jurisdiction's greater stringency or precaution is sample selection bias: selectively citing prominent examples that draw media attention but that do not actually represent a general pattern or trend. A broader perspective is needed to represent the actual pattern of regulatory similarities and differences” (IRGC 2017b).

The IRGC report found different patterns across and within the four key sectors it studied. The variation within each sector further demonstrated the complexity and selectivity of risk regulatory approaches.^20^

- Food: The IRGC report found that European regulations were more stringent regarding genetically modified foods, hormones in beef, and antibiotics in animal production; US regulations were more stringent regarding mad cow disease (BSE/vCJD) (in beef and in blood), trans fats (in labeling and phaseout), unpasteurized dairy products (such as cheeses), and choking hazards (such as “surprise” toys encased in candy); the oft‐cited case of “chlorine‐washed chicken” exhibited greater EU precaution against chlorine byproducts, but also greater US precaution against salmonella exposure; and the case of organic food exhibited policy convergence achieved through international regulatory cooperation.
- Automobiles: The IRGC report found differences in US and EU technical standards for vehicle safety, but similar safety outcomes; more stringent US standards (and more vigorous US enforcement) to control automobile air pollutant emissions (especially NOx and PM2.5), while EU policies promoted greater use of diesel fuels that reduce CO_2_ emissions but may increase conventional air pollutants; and a new opportunity for US‐EU regulatory cooperation in regulating automated vehicles (self‐driving cars and trucks).
- Chemicals: The IRGC report found complex and evolving differences in testing, evaluation, and standard‐setting, under the US Toxics Substances Control Act (TSCA) of 1976, as amended by the Lautenberg Chemical Safety Act (LCSA) of 2016 (which had just been enacted when the IRGC was preparing its report), and European REACH policy implemented since 2006.
- Pharmaceuticals: The IRGC report found that the US and EU have been converging in their approaches to drug licensing, with both moving toward adaptive licensing approaches that enable initial access to needed drugs in subpopulations and then monitor such early access to evaluate iteratively whether broader access is warranted for additional groups; and the report observed differences in drug reimbursement payment systems by governments and insurers.

The report concluded with observations on overall regulatory comparisons, and on incorporating comparative learning into planned adaptive regulation. It summarized its empirical findings of complexity and particularity:

“The reality of transatlantic regulation is not a simple dichotomy of a European approach versus an American approach. It is not EU precaution versus US reaction, or ex‐ante versus ex‐post legal systems, or civil law versus common law, or uncertainty‐based versus evidence‐based regulatory systems. Rather, the reality is overall EU‐US parity as well as some particular variation in policies on both sides of the Atlantic. This includes both cases of greater European stringency and cases of greater US stringency. The EU and US can learn from this variation, and from evolving understanding, to improve regulatory standards through monitoring, evaluation, impact assessment, and planned adaptive regulation.”^21^

In contrast to popular suggestions that a leading regulator representing a large market (such as Brussels or Washington) can effectively set standards that drive global adherence, the IRGC report observed that “Industry may respond to regulatory differences by producing different products to meet different standards in different jurisdictions, or by producing a single product that meets the most stringent standard, or by exiting the product market. This choice is highly sensitive to the costs of each production process, and there does not seem to be a common pattern” (IRGC 2017b).

The IRGC report emphasized that understanding regulatory differences can promote learning and policy improvement. It argued that:

“variation … across risk regulations in the US and Europe is not always a problem: it can also be an important source of learning to inform better future choices. International regulatory cooperation aimed at reducing barriers to trade (via mutual recognition, harmonization, or other modes) begs the question of which standard to recognize or converge on. Studying observed regulatory variation, and even experimentation, can assess differences in outcomes from different regulatory approaches, better choices among current standards, and new approaches not yet adopted by either side. Both the US and Europe could benefit from such policy learning – to increase benefits, lower costs and avoid ancillary harms” (IRGC 2017b).

To enable such policy learning from regulatory variation, the IRGC report recommended careful analysis and international cooperation to collect data, structure comparisons, and evaluate results through retrospective impact assessments (IRGC 2017b).

Further, the IRGC report urged the US and EU to advance “planned adaptive regulation” (PAR), in which each regulation is not only reviewed retrospectively, but is designed from its start to collect data, to learn from experience, and to update over time. PAR responds to uncertainty about the future effects of a regulation by designing an iterative process of learning and adaptive improvements. PAR thus enables governments to take into account evolving evidence on the actual effects of their rules. PAR can be another key mechanism for policy learning—not only from regulatory variation across countries, but also from experience over time—to improve regulatory designs and outcomes.^22^ PAR will be most beneficial when rapid changes (in technologies, scientific understanding, and social conditions) present opportunities for learning and updating, but it can also involve costs, such as the cost of data monitoring and the cost of policy instability; hence, the optimal use of PAR (and the optimal time interval for periodically assessing data and policy revisions) will vary across cases, and its best applications should be carefully selected.^23^

## 3 Impacts From IRGC's Work

IRGC has had an impact on both risk‐related research and policy, but as with all such organizations, moving beyond anecdotal evidence to measuring that impact has been difficult.^24^

With respect to research, IRGC has encouraged and supported interdisciplinary research and stressed that it is important to consider social science in addition to natural science and engineering when it comes to assessing risks. Today, this is increasingly common, but when and how to articulate technical risk assessment and assessment of values, motivations, and preferences (what drives institutional and people's choices) has been efficiently structured in the risk governance framework. The framework is cited by numerous professional guidelines for risk assessment and governance, depending on specific research domains. In China, for example, IRGC's framework has been used in studies on issues ranging from environmental pollution to public health, food safety, urban governance, natural disaster management, and campus bullying. Citations of IRGC's generic framework and guidelines remained at a constant high level over the years.

With respect to policy, IRGC has inspired policy advisors, primarily through international organizations such as the OECD or the EU which organize collaboration among them. Those entities seek guidance and recommendations for how to improve national and international policymaking involving risk, and they often transpose IRGC's advice to their specificities. While IRGC was a source of knowledge and inspiration, also because it wrote for an informed but not expert audience, its best impact was through the “appropriation” process that every policy advisor goes through. A former OECD director spoke of IRGC as “the scout before the caravan.” He meant that IRGC may not have provided all what is needed for direct implementation in policy or regulation, but it provides guidance to those whose job is to write and implement policy around risk or emerging technology‐related risks. Involving policymakers in IRGC's board also had a direct impact. For example, Mr. Liu Yanhua, who served as a board member of IRGC when he was also a Vice Minister of the Chinese Ministry of Science and Technology, supported risk analysis work in academia and adopted IRGC's approach to examine climate risks when he later served as the chair of China's Climate Change Expert Committee.

IRGC has significantly contributed to placing the field of risk governance on research and policy agendas, showcasing that ignoring emerging risks in the first place often explains why technologies are not implemented the way they were expected to develop, or why risks cannot be “fixed” through conventional risk management measures.

## 4 Discussion and Conclusion

IRGC acted as a convening platform to discuss and facilitate the emergence of governance arrangements to develop science‐based and acceptable policy options for handling many risk issues where technology played some role (either as causing or as mitigating risks). Over the course of its 20 years of operation, IRGC organized many meetings and topical workshops and published many reports and concept notes. In the years to come, other organizations will perform similar roles. However, there are three key roles that IRGC provided, which to date, no other organization appears to have filled:

- Providing a neutral international convening platform to facilitate the discussion of important ongoing and emerging technology‐based risks while accounting for a diversity of cultures (political, societal, and regulatory) to develop risk policies that make sense to as many key parties as possible.^25^
- Maintaining a vigorous and engaged network of experts working on all aspects of risk in North America, across the EU, in China, and elsewhere, which is becoming increasingly challenging amidst growing geopolitical complexity.
- Focusing on potential risk issues that were being neglected in research and policy domains to reveal risks that would materialize later in time or elsewhere, even if, at the time of its work, IRGC was perceived as slowing technological innovation.^26^ IRGC's attitude was always to explore, anticipate, and help prevent and prepare, to ensure that risks are taken into account before it is too late for effective action.^27^

It was largely to sustain those functions that many of the coauthors of this paper devoted years of effort to nurturing and sustaining the organization.

When IRGC leadership concluded that there was no viable way to continue its activities, the Board considered several other organizations working on risk‐related issues to which remaining resources could be transferred, and the accumulated intellectual properties and publications could be maintained. The board decided to make this transfer to the Laboratory for Energy Systems Analysis at PSI, which was exploring risks related to transportation in the energy transition. This group is committed to sustaining a connection among the members of the IRGC international network by asking them periodically to serve as advisors for their ongoing work on the energy transition.

While a few faculty at EPFL and ETHZ continue to have risk‐related interests and pursue risk‐related activities, the risk centers at both EPFL and ETHZ have now also been closed. Several other organizations in Switzerland address some risk‐related issues,^28^ but none has the broad domain and international focus of IRGC. While it is too early to see how much of IRGC's networking role it might replace, a new international networking activity among “risk thinkers and doers” has been organized by the ASRA (The Accelerator for Systematic Risk Assessment), which currently develops a framework for systemic risk assessment and response, elaborating on IRGC's work.^29^ Another new effort is the Global Risk Report (expected June 30, 2025) being developed by the United Nations, complementing other global risk reports and ranking exercises.

In the future, one or more of these, or some other risk‐focused organization elsewhere in the world, might offer a risk‐focused neutral international convening platform, similar to what the IRGC provided. However, none has yet filled that space. And, of course, there is also the possibility that IRGC could be reactivated by a new generation of risk experts as a neutral convening organization.

## Data Availability

The data that support the findings of this study are openly available in the International Risk Governance Council at https://irgc.org/

## Endnotes

1 IRGC focused on risks to health, safety, environment, and security, rather than risks that are primarily economic or social.

2 The word “innovation” was added to SERI after IRGC was established.

3 The others were focused on the governance of the risks associated with nanotechnology and with critical infrastructures. Both projects concluded with the publication of IRGC White Papers and Policy Briefs (see Table 1) Attention was also devoted to conferences and the assessment of emerging risks.

4 Renn later became scientific director of the International Institute for Advanced Sustainability Studies (IASS) in Potsdam. See: https://www.uni‐stuttgart.de/en/press/experts/Prof.‐Dr.‐Ortwin‐Renn/.

5 See also: https://irgc.org/wp‐content/uploads/2018/09/Assessing‐Risks1.jpg.

6 The ETH domain, established under Swiss law and controlled by the Federal government, comprises the two Federal Institutes of Technology (ETHZ and EPFL), four Federal research institutes (PSI, EMPA, EAWAG, and WSL), and four topically focused “competence centers.”

7 Many of the experts who were members of this network were also members of the Society for Risk Analysis (SRA and SRA‐E), as well as several other risk and decision‐making professional societies.

8 IRGC publications are available on both https://irgc.org and https://irgc.epfl.ch.

9 In addition to the publications listed in Tables 1 and 2, a Google search for many of these topics, combined with the phrase “IRGC,” will find details on related IRGC activities.

10 As of this writing, most experts argue that such a situation has not yet obtained.

11 A summary of IRGC activities involving SMRs is at: https://irgc.org/issues/energy/small‐modular‐reactors/.

12 For details, see: https://www.epfl.ch/research/domains/irgc/specific‐risk‐domains/synthetic‐biology/.

13 Proceedings available at: https://www.epfl.ch/research/domains/irgc/wp‐content/uploads/2019/11/IRGC‐2019.‐Security‐for‐Emerging‐Synthetic‐Biology‐and‐Biotechnology‐Threats‐Workshop‐report.pdf.

14 See also: https://irgc.org/irgc‐international‐conference‐planning‐adaptive‐risk‐regulation/.

15 See: https://irgc.org/risk‐and‐opportunity‐governance‐of‐autonomous‐cars/.

16
https://www.epfl.ch/research/domains/irgc/specific‐risk‐domains/digitalisation/events‐workshops‐cybersecurityiot‐nov2016/.

17 For details on the resource guides on resilience and other IRGC work in this area, see:https://irgc.org/risk‐governance/resilience/irgc‐resource‐guide‐on‐resilience/.

18 See also: https://irgc.org/risk‐governance/systemic‐risks/guidelines‐governance‐systemic‐risks‐context‐transitions/.

19 See IRGC's spotlight on risk article “Covid‐19: a risk governance perspective”
https://www.epfl.ch/research/domains/irgc/spotlight‐on‐risk‐series/covid‐19‐a‐risk‐governance‐perspective/.

20 See IRGC 2017b (Summary pp. 5−6, and Conclusions Section 3.2 pp. 55−56; and the case studies in Section 2 pp. 15−52).

21 A subsequent study by Li, Xu, and Wiener (2022) undertook a similar comparison of risk regulatory variations across the US and China, and likewise found a complex pattern of selective applications of relative stringency and precaution on each side.

22 See IRGC (2017b), Section 3.5 pp. 58−61.

23 For further discussion of planned adaptive regulation and its best uses, see McCray, Oye, and Petersen (2010), Bennear and Wiener (2019), and Wiener (2020).

24 IRGC's impact was described in some details in its annual reports, available from https://www.epfl.ch/research/domains/irgc/about/what‐we‐do/.

25 One limitation that IRGC faced was difficulty in becoming involved with advising on trans‐boundary and trans‐sectoral risks, where different risk management institutions are involved and where cooperation is required between different actors, or being invited to offer advice at the national level, where many regulations are developed and implemented.

26 This was particularly true for the case of global AI governance, where some technical experts felt the current regulations on AI risks were slowing down innovations.

27 This was the prime motivation for IRGC's last large project work, regarding ways to ensure that emerging technology outcomes would not cause risks to environmental sustainability. See https://www.epfl.ch/research/domains/irgc/eset/.

28 See, for example: https://www.simoninstitute.ch; https://www.risiko‐dialog.ch; https://www.unige.ch/sciences/terre/CERG‐C/.

29 See https://www.asranetwork.org.
